# New genus and species of sisyrids (Insecta, Neuroptera) from the Late Cretaceous Myanmar amber

**DOI:** 10.3897/zookeys.739.22310

**Published:** 2018-02-23

**Authors:** Qiang Yang, Chaofan Shi, Dong Ren, Yongjie Wang, Hong Pang

**Affiliations:** 1 State Key Laboratory of Biocontrol, Key Laboratory of Biodiversity Dynamics and Conservation of Guangdong Higher Education Institute, Ecology and Evolution, School of Life Sciences, Sun Yat-sen University, Guangzhou 510275, PR China; 2 College of Life Sciences, Capital Normal University, Xisanhuanbeilu 105, Haidian District, Beijing 100048, PR China; 3 Geoscience Museum, Hebei GEO University, 136 Huaiandonglu, Shijiazhuang, 050031, PR China; 4 School of Earth Sciences and Engineering, Sun Yat-sen University, Guangzhou 510275, PR China

**Keywords:** spongillaflies, Burmese, Mesozoic, fossil, color spots

## Abstract

A new genus and species of Sisyridae, *Stictosisyra pennyi* **gen. et sp. n.**, is described from the Late Cretaceous (earliest Cenomanian/late Albian) Myanmar amber. It can be easily distinguished from other sisyrids genera by the configuration of wing venation such as forewing with four ra-rp crossveins, M forked distal to the separation of RP1, CuA pectinate and CuP simple; hind wing 1r-m long and sinuous. Besides, the newly documented spongillaflies bore distinct, irregularly distributed spots on the forewings.

## Introduction

Sisyridae is a small and one of the most ancient family of Neuroptera, comprising about 70 extant species (Oswald 2015) assigned to four genera (*Sisyra* Burmeister, 1839, *Sisyrina* Banks, 1939, *Sisyborina* Monserrat, 1981, and *Climacia* McLachlan, 1869). *Sisyra* is cosmopolitan. *Climacia* are distributed in the Nearctic and Neotropics. *Sisyrina* are in the Afrotropics, Indomalaya and Australasia. *Sisyborina* is restricted to the Afrotropics (Oswald 2015). The fossil record of Sisyridae is very rare, dating back to the Late Cretaceous. To date, three extinct genera with six species have been described, all from Eurasia: *Paleosisyra eocenica* Nel, Menier,Waller, Hodebert & de Ploëg, 2003; *P. electrobaltica* Wichard, Gröhn & Seredszus, 2009; *P. minor* Wichard, Wedmann & Weiterschan, 2016; *Prosisyrina sukachevae* Perkovsky & Makarkin, 2015; *P. sphinga* Makarkin & Perkovsky, 2016; and *Paradoxosisyra groehni* Makarkin, 2016. Also Perkovsky and Makarkin (2016) give a list of known fossil Sisyridae including undetermined species.

Here, we describe a new genus and species, *Stictosisyra pennyi* gen. et sp. n., of Sisyridae, from the lowermost Cenomanian Myanmar amber. This is the second species of the family in this locality. The former described species, *Paradoxosisyra groehni*, possessing relatively long, siphonate mouthparts, is unique among Sisyridae. Based on this species, the extinct subfamily Paradoxosisyrinae was erected. Paradoxosisyrinae are common in Burmese amber, with tens of specimens found (pers. obs.). The new genus and species described here does not belong to Paradoxosisyrinae, but shares more characters in common with the extant Sisyridae.

## Material and methods

This study is based on one male specimen from Myanmar amber. The amber pieces were collected in the Hukawng Valley (the state of Kachin in northern Myanmar). A map of the Hukawng Valley is given by Grimaldi et al. (2002, fig. 1). The volcaniclastic matrix of the amber is estimated to be ~98.79 ± 0.62 million years old, i.e., near the Albian/Cenomanian (Early/Late Cretaceous) boundary (Shi et al. 2012). The biological inclusions of Myanmar amber represent a sample of a tropical forest community in equatorial southeastern Asia at ~12_N paleolatitude (Grimaldi et al. 2002; Poinar et al. 2008; Zhang et al. 2017; Ren et al. 2017). The specimen is housed in the collection of the Key Laboratory of Insect Evolution & Environmental Changes, College of Life Sciences, Capital Normal University, Beijing, China (CNUB; Dong Ren, Curator). The specimen was examined using a Zeiss Discovery V20 stereomicroscope and photographed with an AxioCam HRc digital camera attached to the Zeiss Discovery V20 stereomicroscope (both instruments Carl Zeiss Light Microscopy, Göttingen, Germany). Line drawings were prepared with the CorelDraw X4 graphics software and with the aid of Adobe Photoshop CC.

Venation terminology in general follows Kukalová-Peck and Lawrence (2004) as interpreted by Yang et al. (2012, 2014). Terminological details of venation (e.g., spaces, veinlets, traces) follows Oswald (1993). Crossveins are designated after the longitudinal veins with which they connect and are numbered in sequence from the wing base, e.g., 1r-m, a crossvein connecting R/RP and M/MA in the first series.

Abbreviations: AA1–AA3, first to third anterior anal vein; CuA, anterior cubitus; CuP, posterior cubitus; MA and MP, anterior and posterior branches of media; RA, anterior radius; RP, posterior sector; RP1, proximal-most branch of RP; RP2, branch of RP distad RP1; ScA, subcosta anterior; ScP, subcosta posterior.

## Systematic paleontology

### Class Insecta Linnaeus, 1758

#### Order Neuroptera Linnaeus, 1758

##### Family Sisyridae Banks, 1905

###### Genus Stictosisyra

Taxon classificationAnimaliaNeuropteraSisyridae

Yang, Shi, Wang & Pang gen. n.

http://zoobank.org/DD782A02-2AA7-4578-A18C-10F8CBE41B9F

####### Type and only species.

*Stictosisyra pennyi* Yang, Shi, Wang & Pang, gen. et sp. n.

**Etymology.** The generic name is a combination of *stict*- (Greek, meaning speckled, flecky), in reference to the irregular brown spots distributed on the forewings; and *Sisyra*, type genus of the family. Gender feminine.

####### Diagnosis.

Forewing with four ra-rp crossveins, M forked distal to the separation of RP1, CuA pectinately branched, CuA branches simple, CuP simple. Forewing with irregularly distributed spots over whole wing. Hind wing with one ra-rp, 1r-m very long and sinuous, connected stem of RP and M.

###### Stictosisyra pennyi

Taxon classificationAnimaliaNeuropteraSisyridae

Yang, Shi, Wang & Pang sp. n.

http://zoobank.org/C0203F16-A571-4B38-AD51-61593094D8F8

Fig. 1 , 2 , 3

####### Etymology.

The specific epithet is in memory of Dr. Norman D. Penny (1946–2016), in recognition of his great contribution to the lacewing study. The first two authors were impressed by his kindles and generosity when visiting the California Academy of Sciences in 2016.

####### Diagnosis.

As for the genus.

####### Holotype.

CNU-NEU-MA2017006, assumed male, nearly complete and well preserved specimen.

####### Locality and horizon.

Hukawng Valley, Kachin State, northern Myanmar; lowermost Cenomanian, Upper Cretaceous.

####### Description.

Holotype CNU-NEU-MA2017006. Total body length 2.9 mm. Head and body with numerous scattered, fine setae; head about as wide as long. Compound eyes large. Antenna moniliform, with scattered setae all over; scape nearly 2 times as long as wide, slightly thicker than flagellum; pedicel elongate, about 2 times as long as wide; flagellum with about 25 flagellomeres, first flagellomere longer than others, about as long as pedicel, and last one elongate, about 2.5 times as long as other flagellomeres, apically tapered. Pronotum narrower than head, twice as long as wide; pro-, meso- and metanotum with scattered, fine, long setae. Legs relatively long and slender, with numerous short setae intermixed with long setae. Foreleg: coxa elongated; femur long and slender; tibia nearly as long as femur; basitarsus nearly thrice as long as second tarsomere, the last four tarsomeres of same length. Mid-, hind legs poorly preserved. Abdomen nine segments, with scattered short setae.

Forewing length 3.0 mm, width 1.1 mm (right forewing); elongated ovoid, apex rounded, with dense relatively short setae on veins and longer setae on margins; membrane with fuscous spots over whole wing; trichosors prominent along entire wing margin. Humeral vein short and simple, not recurrent, perpendicular to ScP; presumable ScA not detected; costal space narrow; subcostal veinlets simple, not forked, pterostigma not present. ScP and RA fused distally, entering margin before wing apex. Only one sc-r present. Four ra-rp crossveins, distalmost ra-rp crossvein located at fusion of ScP and RA; RP separated from RA just proximal to sc-r, with three branches. RP1, RP2 configuration similarly, dichotomously forked, RP3 with a distal fork, about two crossveins between RP1, RP2, and one crossvein between RP2, RP3. M divided into MA and MP at 2m-cu, far from separation of RP1 from RP stem, one ma-mp crossvein present; MA distally forked twice, MP pectinately forked, with two branches distally; three r-m crossveins between RP and M; Cu divided into CuA and CuP near wing base at level of RP origin, three m-cu crossveins; CuA pectinately forked, with three (right forewing) or four (left forewing) simple branches distal to 2m-cu; CuP simple, one crossvein between CuA, CuP; only one 2cu-aa visible; AA1, AA2, AA3 configuration similarly, each with a distal fork, no crossveins detected between AA region.

Hind wing elongate, slightly smaller than forewing, length 2.5 mm, width 0.9 mm (right hind wing). Trichosors prominent along entire wing margin. No color spots on wing or along margin. Costal space narrow, distally dilated, especially distad fusion of ScP and RA. Subcostal veinlets simple, sparsely spaced, pterostigma not present. Subcostal space broader than costal space, basally narrowed; no crossvein detected. ScP, RA fused distally. ScP+RA entering wing margin before apex, with three simple distal veinlets. RA space wider than subcostal space, with one crossveins located between origin of RP2 and RP3. RP originated near wing base, with three branches originating far from wing base, each forked distally. Stem of RP and RP3 just with a distal fork; RP1 dichomously forked, RP2 forked twice distally. Three crossvein between RP region; three r-m crossveins between RP and M, basal 1r-m between stem R and M long and strongly sinuous. M forked distad origin of RP and proximal to origin of RP1. MA dichotomously branched distally; MP forked twice distally. Only one crossvein between MA and MP. CuA long, pectinately branched with about three simple branches; CuP long and simple. One crossvein between M and Cu; one crossvein visible between CuA and CuP. Anal veins not preserved.

**Figure 1.**
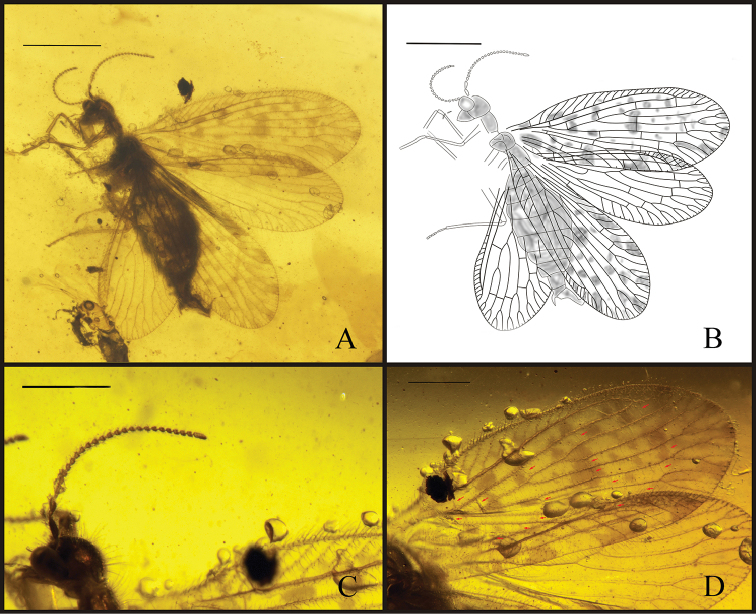
*Stictosisyra pennyi* gen. et sp. n., holotype CNU-NEU-MA2017006. **A** photograph of holotype **B** line drawing of holotype **C** detail photograph of antenna **D** forewing with a squint view, red arrow shows the distribution of crossveins. Scale bars: 1 mm (**A, B**); 0.5mm (**C, D**).

**Figure 2.**
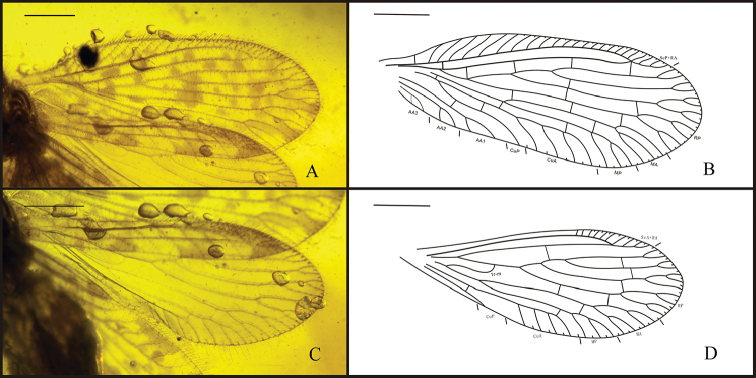
*Stictosisyra pennyi* gen. et sp. n., holotype CNU-NEU-MA2017006. **A** photograph of forewing **B** line drawing of forewing **C** photograph of hind wing **D** line drawing of hind wing. Scale bars: 0.5 mm.

**Figure 3.**
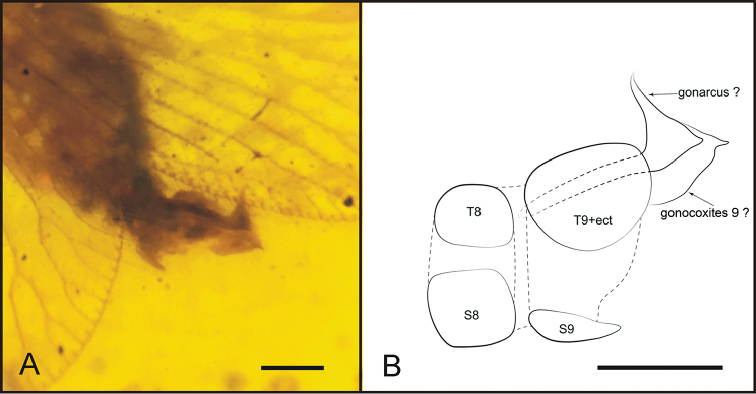
*Stictosisyra pennyi* gen. et sp. n., holotype CNU-NEU-MA2017006. **A** photograph of genitalia **B** line drawing of genitalia (T: tergite; S: sternite; ect: ectoproct). Scale bars: 0.2 mm.

####### Remarks.

*Stictosisyra* gen. n. is different from other fossil sisyrids genera in the following characters: 1) without long siphonate mouthparts (*Paradoxosisyra* with long siphonate mouthparts); 2) forewing with four ra-rp (*Paleosisyra eocenica* with two, *P. electrobaltica* and *P. minor* with three); 3) hind wing ScP and RA fused before entering the margin, and with one ra-rp (*Prosisyrina* hind wing ScP and RA terminate separately, with two ra-rp).

####### Comments.

***Male genitalia.*** The genitalia of the holotype was not well preserved in the amber; furthermore, the morphology is quite different from that of other sisyrids. Herein we tentatively assume the specimen to be a male based on the morphology of abdomen, especially of the terminalia, and the preserved posture. The genitalia are interpreted as follows: tergite 9 and ectoproct fused; sternite 9 shorter than tergite 9+ectoproct; gonarcus extend beyond tergite 9+ectoproct, otherwise, may be caused by posteriorly incomplete preservation of tergite 9+ectoproct; gonarcus narrowly arched medially, with two arms ventrally and afterwards anteriorly extended; each arm with a tiny extension pointed posteriorly; large, and almost whole external gonocoxites 9, connected with gonarcus.

## Supplementary Material

XML Treatment for Stictosisyra

XML Treatment for Stictosisyra pennyi
